# Dynamic Changes in Gut Microbiota-Derived Metabolite Trimethylamine-N-Oxide and Risk of Type 2 Diabetes Mellitus: Potential for Dietary Changes in Diabetes Prevention

**DOI:** 10.3390/nu16111711

**Published:** 2024-05-30

**Authors:** Yuliang Huang, Yani Wu, Yao Zhang, He Bai, Ruiheng Peng, Wenli Ruan, Qianlong Zhang, Enmao Cai, Mingfeng Ma, Yueyang Zhao, Ying Lu, Liqiang Zheng

**Affiliations:** 1 Department of Acute Communicable Diseases Control and Prevention, Huangpu District Center for Disease Control and Prevention, Shanghai 200023, China; huangyuliang@hpcdc.sh.cn; 2 School of Public Health, Shanghai Jiao Tong University School of Medicine, Shanghai 200025, China; soulnsn@sjtu.edu.cn (Y.W.); bh17737151922@163.com (H.B.); pengruiheng@sjtu.edu.cn (R.P.); 3 Department of Endocrinology, Shengjing Hospital of China Medical University, Shenyang 110004, China; 18940258302@163.com; 4 Department of Physical and Chemical, Changning District Center for Disease Control and Prevention, Shanghai 200051, China; rwlyexiu@sina.com (W.R.); caienmao@126.com (E.C.); 5 Ministry of Education-Shanghai Key Laboratory of Children’s Environmental Health, Xinhua Hospital, Shanghai Jiao Tong University School of Medicine, Shanghai 200092, China; zhangql7989@163.com; 6 Department of Cardiovascular Medicine, Fenyang Hospital, Shanxi Medical University, Fenyang 032200, China; mamingfeng106@sina.com; 7 Library, Shengjing Hospital of China Medical University, Shenyang 110004, China

**Keywords:** type 2 diabetes mellitus, T2DM, trimethylamine N-oxide, TMAO, prospective cohort, risk factors

## Abstract

Background: A gut-microbial metabolite, trimethylamine N-oxide (TMAO), has been associated with type 2 diabetes mellitus (T2DM). Few previous prospective studies have addressed associations between the changes in TMAO and T2DM incidence. Methods: Data were derived from a longitudinal cohort conducted from 2019 to 2021 in rural areas of Fuxin County, Liaoning Province, China, and 1515 diabetes-free participants aged above 35 years were included. The concentrations of serum TMAO and its precursors were measured at two time points, namely in 2019 and 2021. TMAO and TMAO changes (ΔTMAO) were separately tested in a logistic regression model. For further examination, the odds ratios (ORs) for T2DM were calculated according to a combination of TMAO levels and ΔTMAO levels. Results: During a median follow-up of 1.85 years, 81 incident cases of T2DM (5.35%) were identified. Baseline TMAO levels exhibited a nonlinear relationship, first decreasing and then increasing, and only at the highest quartile was it associated with the risk of T2DM. The OR for T2DM in the highest quartile of serum TMAO was 3.35 (95%CI: 1.55–7.26, *p* = 0.002), compared with the lowest quartile. As for its precursors, only choline level was associated with T2DM risk and the OR for T2DM in the Q3 and Q4 of serum choline was 3.37 (95%CI: 1.41–8.05, *p* = 0.006) and 4.72 (95%CI: 1.47–15.13, *p* = 0.009), respectively. When considering both baseline TMAO levels and ΔTMAO over time, participants with sustained high TMAO levels demonstrated a significantly increased risk of T2DM, with a multivariable-adjusted OR of 8.68 (95%CI: 1.97, 38.34). Conclusion: Both initial serum TMAO levels and long-term serum TMAO changes were collectively and significantly associated with the occurrence of subsequent T2DM events. Interventions aimed at normalizing TMAO levels, such as adopting a healthy dietary pattern, may be particularly beneficial in T2DM prevention.

## 1. Introduction

Type 2 diabetes mellitus (T2DM) is a serious public health challenge associated with elevated morbidity and mortality rates, imposing a substantial health and economic burden [1]. Consequently, the prevention of T2DM is a recognized public health priority. Lifestyle factors play a crucial role in both the occurrence and progression of T2DM and studies have indicated that diet and gut microbiota may impact T2DM [2].

Trimethylamine N-oxide (TMAO), an organic compound of amine oxides with the molecular formula (CH3)3NO, is the most focused metabolite originating from intestinal microorganisms [3,4]. It can affect the structure and activity of many biologically important compounds [5]. TMAO primarily stems from dietary sources rich in choline, carnitine, and betaine, such as red meat, eggs, dairy products, and saltwater fish [6]. Choline, carnitine, and betaine are metabolized to produce trimethylamine (TMA) via microbial enzymes. TMA is absorbed by intestinal capillaries and subsequently transported into the portal circulation, ultimately reaching the liver. Within the hepatic environment, the enzymatic action of flavin monooxygenase 3 (FMO3) facilitates the conversion of TMA into TMAO [7]. About 50% of TMAO in the human body is not metabolized; the vast majority is excreted in the urine by the kidneys and some is reduced to TMA by bacteria and excreted in the feces or respiration [8,9].

TMAO has been identified as a novel independent risk factor associated with atherosclerosis and cardiovascular disease (CVD) [10,11], exhibiting a dose–response relationship with plasma levels and the risk of developing CVD. This metabolite may serve as a potential link connecting dietary patterns, gut microbiota, and the development of lifestyle-related diseases. Experimental animal models have demonstrated that TMAO can cause adipose tissue inflammation and disrupt the insulin signaling pathway, which can lead to insulin resistance and promote the development of diabetes [12,13]. TMAO plays an important role in the occurrence, development, and related complications of diabetes [14].

A recent meta-analysis [14] has underscored a positive, dose-dependent correlation between circulating TMAO levels and heightened risk of diabetes. However, clinical investigations on TMAO and T2DM remain limited, with the bulk of the literature included in meta-analyses predominantly focusing on the role of TMAO in cardiovascular diseases, potentially introducing heterogeneity. While many observational studies have found a significant association between serum TMAO and the risk of T2DM [14,15,16], some studies have found no relationship between the two [17,18], and even the opposite conclusion [19] has been observed. Some factors, such as ethnicity, sample size, or diet patterns in different areas, might have caused those inconsistent results. On the other hand, most of these studies have a cross-sectional design, with only a minority being longitudinal cohort studies. Given the limitations of cross-sectional studies, the lack of longitudinal, prospective data on this topic constitutes an important gap in the literature.

Notably, previous studies that investigated the associations between TMAO and T2DM risk solely relied on data collected at a single time point. However, analyzing dynamic changes in risk factors may offer more relevant insights for translating findings into preventive strategies [20]. Assessing the temporal dynamics of TMAO levels would provide valuable insights into the modifiability of circulating TMAO concerning the risk of subsequent T2DM events. Such an analysis would be instrumental in advancing the development of novel intervention strategies aimed at preventing T2DM. Therefore, in this large-sample prospective cohort with a follow-up of 1.85 years, we measured serum concentrations of the gut-microbial metabolite TMAO and its precursor substances, including choline, carnitine, and betaine at two time points, among the rural population in northern China. We aimed to investigate whether the concentrations of TMAO and its precursors, as well as the longitudinal changes in their concentration levels, are associated with the subsequent T2DM incidence.

## 2. Methods

### 2.1. Study Population

The data for this study were obtained from a longitudinal cohort study conducted in rural areas of Fuxin Autonomous County, Liaoning Province, China. Fuxin, located in the northeast region of China, exhibits a dietary pattern characterized by a high consumption of flour and its products, miscellaneous grains and tubers, and eggs. Due to its geographical location and lower rural economic level, seafood intake is generally low. Furthermore, the overall cultural level in the region limits the awareness of healthy living practices. To ensure sample representativeness, the research area was divided into three parts: eastern, southern, and northern. Based on demographic characteristics, two townships from the southern part, one from the northern part, and one from the eastern part were selected, respectively. Thirty-three villages were then chosen from these four townships based on geographic locations. A questionnaire survey of the general population was conducted from June 2019 to August 2019. Participants were considered eligible if they were 35 years of age or older, had stayed in the study area for at least five years, and were willing to sign a consent form. Exclusions were made for individuals who were pregnant, had severe liver or renal failure, or were unwilling to participate. Ultimately, 4689 participants were recruited for the study. The follow-up survey was conducted from June 2021 to August 2021, with the same inclusion and exclusion criteria applied at baseline. Blood samples were provided at these two time points.

For the present study, we excluded participants according to our prespecified criteria: (1) diabetes at baseline (n = 655); (2) without measurements of TMAO (n = 2384) or fasting glucose (n = 38) at baseline; and (3) other key data missing (n = 2). Then we excluded participants again among the remaining participants according to the following criteria: (1) loss to follow-up (n = 37) and (2) without measurements of TMAO (n = 49) or fasting glucose (n = 9) in the second-wave survey. Finally, 1515 participants were included in the present analyses.

Data on demographics and other factors, including demographic features, lifestyle, and history of disease, were recorded by interview. Written informed consent was obtained from all participants involved in the study. For illiterate participants, written informed consent was obtained from their proxies. The procedures adhered to the ethical standards of the responsible committee on human experimentation at China Medical University (No. [2018]083).

### 2.2. Detection of Serum TMAO and Its Precursors by High-Performance Liquid Chromatography-Tandem Mass Spectrometry

The contents of TMAO and its precursors (choline, betaine, and carnitine) in serum were determined by high-performance liquid chromatography-tandem mass spectrometry (HPLC-MS/MS, Shimadzu, Kyoto, Japan). Then, 10 μL of serum was diluted 50 times with the standard, vortexed and mixed, and then centrifuged at 4 °C and 15,000 r/min for 15 min. The supernatant was taken and passed through a 0.22 μm hydrophobic nylon filter membrane, and finally, 100 μL of solution was transferred to a sealed sample vial. HPLC-MS/MS was used for detection.

Chromatographic conditions: ACQUITY UPLC HSS T3 column (2.1 mm × 100 mm, 1.7 μm), column temperature 40 °C, injection volume 2 μL. Mobile phase A:10 mmol/L ammonium formate, 0.1% formic acid, acetonitrile/water = 9:1; mobile phase B: 5mmol/L ammonium formate, 0.1% formic acid, acetonitrile/water = 1:1. Gradient elution: 0~2.0 min, mobile phase B was 10%; 2.0~6.0 min, from mobile phase B 10% linear change to mobile phase B 45%; 6.0~6.1 min, mobile phase B 45% linear change to mobile phase B 100%; 6.1~8.1 min, mobile phase B 100%; 8.1–8.2 min, from mobile phase B 100% back to mobile phase B 10%; 8.2–10.0 min, mobile phase B 10%. The flow rate was 0.4 mL/min (split ratio 5:3).

Mass spectrometry conditions: Electrospray ionization source (ESI), impact voltage 20eV, positive ion mode, multiple reaction monitoring (MRM), drying gas flow rate 3 L/min, atomizer pressure 50 psi, dryer temperature 350 °C, capillary voltage 3500 V.

Detection effect: When the impact voltage was set to 20 eV and the quantitative ion pair was determined to be m/z 76.1 to 58.1, the detection limit and quantitative limit of this detection method were 0.003 and 0.062 μmol/L, respectively. The linear range was 0.16~20.00 μmol/L (r^2^ = 0.999) and the recovery rate was 90.20~102.10%. The HPLC-MS/MS method for the detection of TMAO established by our research team has the characteristics of being fast, accurate, and sensitive, which could meet the requirements of blood sample detection in large sample populations.

### 2.3. Definition of T2DM Cases

T2DM was defined as fasting plasma glucose ≥7.0 mmol/L (≥126 mg/dL) or self-reporting diabetes diagnosis by a physician or other health professional [21]. Eighty-one T2DM cases were ascertained during a median follow-up of 1.85 years.

### 2.4. Assessment of Other Variables

All surveys were conducted by well-trained local physicians who underwent comprehensive training before initiating the survey. This training encompassed the study’s objectives, questionnaire administration methods, the standard measurement method, the importance of standardization, and the study procedures. After training, rigorous testing was performed, and only those with higher scores could become investigators. The specific survey contents were as follows: gender, age, ethnicity, education, marital status, family per capita income, lifestyle, habitual dietary intakes, disease history, and medication history. Smoking was defined as at least one cigarette per day for more than half a year. Alcohol consumption was defined as at least three drinks per week for more than half a year. Body Mass Index (BMI) = weight/height^2^ (kg/m^2^). Hypertension and hyperlipidemia were defined as self-reports by a physician or other health professional.

Fasting blood samples were collected in the morning from participants who had fasted for at least 8 h. Fasting plasma glucose was measured by using a hexokinase method. Triglyceride and serum uric acid were measured by using colorimetry. Total cholesterol, low-density lipoprotein cholesterol, and high-density lipoprotein cholesterol were measured by using enzyme colorimetry. All laboratory equipment underwent calibration, and the blood samples from all participants were randomly coded and tested blindly. This approach was implemented to effectively minimize systematic error and variability in laboratory batch measurements. The above blood indicators were analyzed using the Roche Cobas 8000 C701 automatic biochemical analyzer in an accredited central laboratory.

### 2.5. Statistical Analysis

Continuous variables were presented as mean ± standard deviation (SD) or median (interquartile range, IQR), and categorical variables were expressed as numbers with percentages. A Student’s *t*-test and Wilcoxon–Mann–Whitney rank sum test were used to compare continuous differences, and the chi-square test was used to compare categorical differences.

Serum TMAO concentration at baseline and serum TMAO concentration changes between 2019 and 2021 (ΔTMAO) were both divided into quartiles. TMAO and ΔTMAO were separately tested in a logistic regression model with the two variables on a continuous scale, with results expressed as an odds ratio (OR) for T2DM as a function of a 1 SD increase on the continuous scale. TMAO and ΔTMAO were also both divided into quartiles, and the lowest quartiles were chosen as the reference in a logistic regression model. To further examine, an OR for T2DM was calculated according to a combination of TMAO levels and ΔTMAO levels. All the logistic regression models were adjusted for age, sex, BMI, current smoking status, current drinking status, diet, hypertension, and hyperlipidemia.

Data were input into Epidata 3.0 software (EpiData Association, Odense, Denmark) and analysis was performed using SPSS, version 27.0 (IBM Corp., Armonk, NY, USA). A two-sided *p* < 0.05 was considered statistically significant.

## 3. Results

In our study, 34.5% of participants were male, with a mean age at baseline of 59.18 ± 9.43 years and a mean BMI of 24.76 ± 3.66 kg/m^2^ across all participants. The median serum TMAO concentration was 4.16 (IQR, 2.19, 7.64) μmol/L in adults over 35 years of age, 4.27 (IQR, 2.07, 7.30) μmol/L in males, and 4.07 (IQR, 2.24, 7.69) μmol/L in females. During a median follow-up of 1.85 years, 81 incident cases of T2DM (5.35%) were identified.

The characteristics of the 1515 participants (81 T2DM cases and 1434 controls) are shown in Table 1. The T2DM cases had a higher BMI, higher fasting plasma glucose, higher triglycerides, higher total cholesterol, and higher HDL cholesterol at baseline. There were no differences in the median values of serum TMAO levels at the first collection between cases and controls (*p* = 0.187), whereas plasma TMAO levels at the second collection were higher in T2DM cases than controls (median values of TMAO: 4.51 (IQR, 1.96, 7.02) µmol/L in cases; 3.18 (IQR, 1.59, 5.91) µmol/L in controls; *p* = 0.005). The serum TMAO concentration at baseline was 4.09 (IQR, 2.17, 7.53) µmol/L for controls and 5.08 (IQR, 2.27, 8.91) µmol/L for cases. The serum TMAO concentration changes between 2019 and 2021 (ΔTMAO) were −0.90 (IQR, −3.96, 1.72) µmol/L for controls and −0.56 (IQR, −3.11, 2.68) µmol/L for cases. For the three precursors of TMAO—choline, betaine, and carnitine—there were no statistically significant differences in serum concentrations between the control and case groups at the first blood collection (*p* > 0.05). However, in the assessment of 2021, serum concentrations of choline and carnitine differed between the two groups, with the case group’s results being significantly lower than those of the control group (median values of betaine: 83.57 (IQR, 66.88, 107.40) µmol/L in cases; 93.38 (IQR, 73.5, 120.55) µmol/L in controls, *p* = 0.047; median values of carnitine: 48.24 (IQR, 40.88, 58.06) µmol/L in cases; 52.25 (IQR, 43.06, 63.16) µmol/L in controls, *p* = 0.026). There were no differences between the two groups in the changes in concentrations of these three precursors from 2019 to 2021.

**Figure 1:**
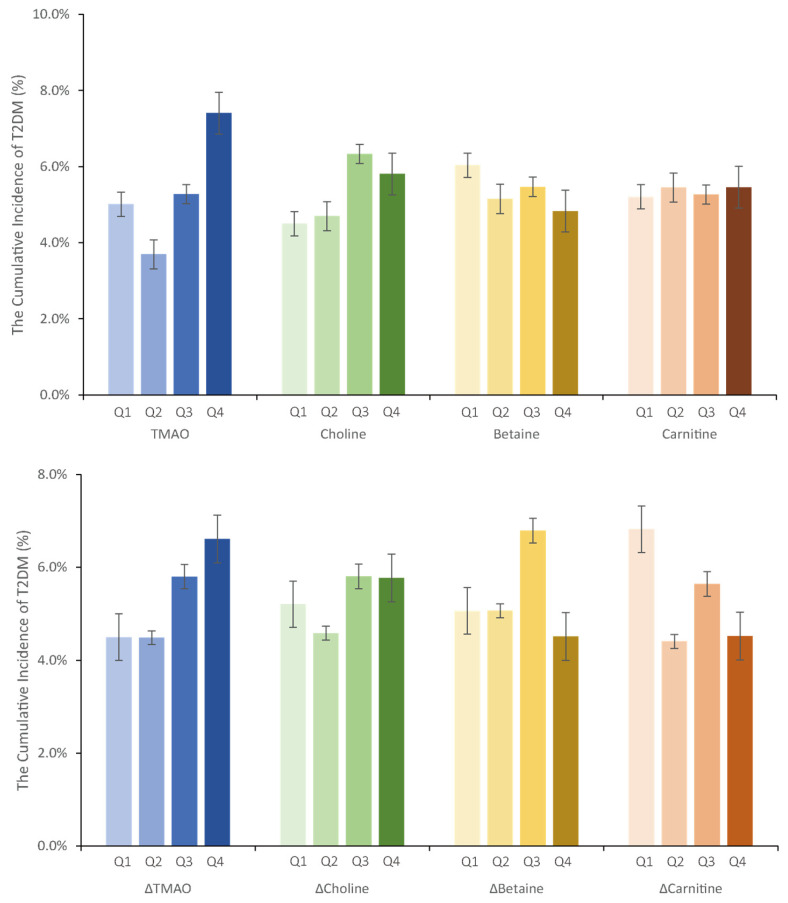
The cumulative incidence of T2DM at different serum substance levels. TMAO levels: Q1 (≤2.19), Q2 (2.19 to 4.16), Q3 (4.16 to 7.64), Q4 (≥7.64). Choline levels: Q1 (≤130.76), Q2 (130.76 to 184.06), Q3 (180.06 to 247.96), Q4 (≥247.96). Betaine levels: Q1 (≤75.39), Q2 (75.39 to 103.70), Q3 (103.70 to 147.59), Q4 (≥147.59). Carnitine levels: Q1 (≤40.09), Q2 (40.09 to 51.18), Q3 (51.18 to 70.90), Q4 (≥70.90). ΔTMAO levels: Q1 (≤−3.95), Q2 (−3.95 to −0.87), Q3 (−0.87 to 1.76), Q4 (≥1.76). ΔCholine levels: Q1 (≤−160.60), Q2 (−160.60 to −87.52), Q3 (−87.52 to −28.19), Q4 (≥−28.19). ΔBetaine levels: Q1 (≤−53.66), Q2 (−53.66 to −12.75), Q3 (−12.75 to 19.94), Q4 (≥19.94). ΔCarnitine levels: Q1 (≤−20.65), Q2 (−20.65 to −1.03), Q3 (−1.03 to 15.05), Q4 (≥15.05). The concentration units of the above substances are all expressed in µmol/L.

Figure 1 illustrates the cumulative incidence of T2DM across different levels of TMAO or its precursors. Upon categorizing TMAO into four quartiles, the cumulative incidences were 5.0%, 3.7%, 5.3%, and 7.4%, respectively (*p* = 0.151). Similarly, when ΔTMAO was stratified into four quartiles, the cumulative incidences were 4.5%, 4.5%, 5.8%, and 6.6%, respectively (*p* = 0.486). In Figure 1, T2DM incidences are calculated based on quartiles of choline, betaine, and carnitine, and changes in precursor levels. Notably, upon further stratification of both TMAO and ΔTMAO into four levels based on low (≤4.159 µmol/L) or high (≥4.160 µmol/L) TMAO levels and low (≤−0.870 µmol/L) or high (≥−0.871 µmol/L) ΔTMAO levels, the cumulative incidences were 1.1%, 5.7%, 5.9%, and 9.8%, respectively (*p* = 0.012).

The associations between baseline serum TMAO and its precursors levels and T2DM risk are presented in Table 2. There were no associations between baseline serum TMAO and T2DM risk in Model 1, which was adjusted for demographic variables including age, sex, BMI, hypertension, and hyperlipidemia, and in Model 2, which encompassed additional adjustments for lifestyle factors such as physical activity, smoking status, drinking status, and dietary habits, including the daily average frequency of consumption of red meat, fish, dairy products, and eggs (*p* > 0.05). In the fully adjusted model further including ΔTMAO (Model 3), the OR for T2DM in the highest quartile of serum TMAO was 3.35 (95% CI: 1.55–7.26, *p* = 0.002), compared with the lowest quartile. Additionally, the OR for T2DM in Q3 and Q4 of serum choline was 3.37 (95% CI: 1.41–8.05, *p* = 0.006) and 4.72 (95% CI: 1.47–15.13, *p* = 0.009), respectively. As for the other two precursors, betaine and carnitine, there were no significant association between their concentration levels at baseline and in T2DM in all models.

Table 3 shows ORs for T2DM according to ΔTMAO and changes in its precursors. In the fully adjusted Model 3, as compared to participants with decreased TMAO levels (Q1 group: ΔTMAO ≤ −3.948 µmol/L), those with the largest increases in TMAO (Q4 group: ΔTMAO ≥ 1.76 µmol/L) had a 3.69 (95% CI: 1.66–8.17, *p* = 0.001)-times higher risk of T2DM. Meanwhile, ΔCholine showed similar results. Participants exhibiting the least reduction in choline (Q4 group: ΔCholine ≥ −28.18 µmol/L) had a 4.56-fold higher risk of T2DM (95% CI: 1.43–14.55, *p* = 0.010) compared to those with greatest decreased choline levels (Q1 group: ΔCholine ≤ −160.60 µmol/L). Every 1 SD increase in ΔCholine was associated with an 80% increased risk for T2DM (OR of per 1 SD: 1.80, 95%CI: 1.11–2.90, *p* = 0.017). For both betaine and carnitine, the concentration changes between the two assessments showed no association with the incidence of T2DM.

We further investigated whether long-term overall TMAO levels were associated with a significantly increased risk of developing T2DM by analyzing the risk according to a combination of baseline TMAO levels and ΔTMAO levels (Table 4). Participants were categorized into four groups based on the Q3–Q4 vs. two lower-quartile (Q1–Q2) cut-offs of TMAO and ΔTMAO levels. Compared to participants with low baseline TMAO levels and low ΔTMAO (Q1–Q2 in both), those with sustained high TMAO levels (in the highest TMAO quartile, Q3–Q4, at baseline and changes) exhibited a significantly increased risk of T2DM, with a multivariable-adjusted OR of 8.68 (95% CI:1.97, 38.34, *p* = 0.004) in the crude model. The association was attenuated after adjusting for demographic factors and lifestyles with an OR of 7.89 (95% CI: 1.77, 35.23, *p* = 0.007) in Model 2. These risks were greater than those observed in individuals with only high baseline TMAO levels or only high ΔTMAO levels.

## 4. Discussion

In this 1.85-year prospective cohort study of 1515 Chinese adults aged over 35 years in a rural area of northeast China, we found a significant association between elevated serum TMAO concentrations and an increased risk of T2DM. Among the three precursors of TMAO—choline, betaine, and carnitine—only choline concentration demonstrated a parallel association with T2DM. Moreover, the present study newly revealed that long-term dynamic changes in the gut-microbial metabolite TMAO in serum were significantly associated with the subsequent T2DM events, independent of the baseline TMAO levels.

The current study breaks new ground by examining dynamic changes in gut-microbial metabolites for T2DM, revealing that evaluating longitudinal alterations in TMAO enhances the identification of individuals at heightened risk of T2DM. These results emphasize the significance of repeated serum TMAO measurements in predicting T2DM risk. Based on repeated measures, people with high levels of TMAO at baseline and a subsequent continuous rise have a significantly increased risk of T2DM, although this trend is attenuated after adjusting for factors such as lifestyle. A similar situation has also emerged in studies of TMAO versus CVD [22]. We observed that baseline TMAO levels exhibited a nonlinear relationship of first decreasing and then increasing, and only at the highest quartile were they associated with the risk of T2DM. As for its precursors, only the choline level was associated with T2DM risk in the third and highest quartile. A community case-control study conducted in China also showed similar results [23]. In another case-control study involving 133 cases, investigating the association between plasma TMAO levels and T2DM within a Saudi Arabian population, it was similarly observed that even after additional adjustments for physical activity and dietary factors, only plasma TMAO levels in the highest quartile (>6.40 μmol/L) were associated with an elevated risk of T2DM [16].

Our findings are supported by previous experimental research that suggests there are several potential mechanisms underlying the association between TMAO and T2DM. Supplementing TMAO in mice fed a high-fat diet exacerbates glucose tolerance, suppresses hepatic insulin signaling pathways, and enhances adipose tissue inflammation. These effects contribute to insulin resistance and the occurrence of diabetes [24]. An animal experiment conducted in mice revealed that TMAO binds to and activates PERK(EIF2AK3) to promote metabolic dysfunction [25]. TMAO triggers the activation of the transcription factor FoxO1, known for its pivotal role in metabolic disorders, through a PERK-dependent pathway. Moreover, strategies aimed at lowering TMAO levels, such as the modulation of gut microbiota or inhibition of the TMAO-synthesizing enzyme FMO3, have been shown to mitigate PERK activation and decrease FoxO1 expression in hepatic tissues. A study reported that TMAO reduces the synthesis of bile acids by inhibiting cholesterol 7α-hydroxylase, thereby decreasing the size of the bile acid pool [26]. Bile acids can improve glucose tolerance and insulin sensitivity through various pathways. Therefore, TMAO may also influence glucose homeostasis by regulating bile acid metabolism. On the other hand, TMAO is also associated with genes involved in the insulin signaling pathway. These genes stimulate the inhibition of liver glycogen synthesis, promote gluconeogenesis, and reduce hepatic glycogen transport capacity [24]. Further studies are needed to elucidate the potential mechanisms of TMAO and diabetes pathogenesis.

An international collaborative project including 16 population-based studies from the United States, Europe, and Asia has shown that circulating TMAO was positively associated with intakes of animal protein, saturated fat, monounsaturated fat, fish, shellfish, eggs, and red meat and inversely associated with intakes of plant protein, carbohydrate, and nuts [27]. The results of current randomized controlled trials have indicated that red meat, as opposed to white meat, increases circulating TMAO levels. Under a Mediterranean dietary pattern, reducing the intake of red meat leads to decreased TMAO concentrations in the circulation. Researchers also revealed three potential mechanisms through which a red meat diet increases circulating TMAO levels: (i) enhanced nutrient density of dietary TMA precursors; (ii) increased microbial TMA/TMAO production; and (iii) reduced renal TMAO excretion [28,29]. Additionally, diet rapidly and reproducibly alters the human gut microbiome [30]. In animal models, a significant reduction in Clostridiales is detected when TMAO levels decrease through dietary regulation [31]. This evidence suggests that diet is one of the most crucial modifiable factors in regulating both circulating TMAO levels and the gut microbiota composition. Given our findings revealing the association between serum TMAO and choline and T2DM, this suggests that dietary restrictions on TMAO and its precursors may improve insulin resistance.

Additionally, as a metabolite of gut microbiota, TMAO’s regulation of gut microbiota and the pathway from TMA to TMAO conversion may also serve as effective methods to reduce TMAO production and thus improve glucose homeostasis. Some studies have revealed gut microbiota associated with circulating TMAO, such as Anaerosporobacter, Clostridiales, Phascolarctobacterium, Oscillibacter, and Alistipes. Among them, Clostridiales has been reported to contain the TMAO metabolism genes Cut C/D [32,33]. A recent systematic review evaluated the impact of diet interventions on the gut microbiota, aiming to uncover potential correlations that could enhance the efficacy of diabetes treatment and management [34]. For example, a randomized controlled trial revealed that high-fiber diets resulted in increased levels of Firmicutes and Bacteroidota, resulting in better insulin control [35]. In patients with T2DM, a diet strategy of medicine-derived food plants and whole grains accompanied by intermittent energy restriction effectively reverses high blood sugar by significantly increasing the abundance of Bacteroidetes, Parabacteroides, and Roseburia [36]. These pieces of evidence provide the possibility that dietary interventions alter gut microbiota and their metabolites. In conclusion, the gut-microbiota metabolite TMAO and its precursors play significant roles in the occurrence and development of T2DM, holding promising applications in the diagnosis and treatment of diabetes mellitus. In the future, lowering TMAO and its precursor levels by altering dietary patterns or intervening in gut microbiota may potentially delay the onset and progression of T2DM. Further experimental and clinical studies are needed to assess the efficacy of different interventions on TMAO levels and their role in preventing the progression or controlling the prognosis of T2DM.

Among the three precursors of TMAO—choline, betaine, and carnitine—only choline concentration demonstrated a parallel association with T2DM. Choline and carnitine are primarily sourced from red meat, poultry, eggs, and dairy products. However, choline is also abundant in fish, shellfish, legumes, and nuts. Consequently, compared to carnitine, choline exhibits a broader spectrum of dietary sources [27,37]. On the other hand, the formation of TMAO is a complex process, which begins with the gut microbiota metabolizing dietary precursors of TMA. This process involves the participation of enzymes such as choline TMA lyase, carnitine oxygenase, and betaine reductase. Interestingly, in non-vegetarian populations, the gene abundance of choline TMA-lyase has been found to be significantly higher than that of carnitine oxygenase [38]. Therefore, the ability of choline metabolism to generate TMAO may be stronger compared to that of carnitine. Notably, in our study, precursors were measured in serum, which may not accurately reflect the concentrations in diet or encountered by bacteria in the gut. This is because these compounds are also absorbed by the host, where they are utilized for energy generation or metabolized into other molecules. Hence, both the concentration and composition of precursors measured in serum probably differ from those taken up by the host through diet, independently of the TMA and TMAO forming process. Therefore, in the context of this study, the associations and applications of precursors with diseases should be carefully and cautiously considered.

Additionally, our study presents one of the largest datasets of TMAO and its precursors reported for the Chinese rural population to date. The overall concentration of TMAO varies widely among studies. It has been shown that the overall concentration of TMAO in normal healthy individuals is in the range of 0.5–5 μmol/L [31], and the medium serum TMAO concentration is about 3 μmol/L [39], which is close to the results of this study. Nie et al. [40] showed that the median serum TMAO concentration in healthy adults aged 45–75 was 2.3 (IQR, 1.4, 3.7) μmol/L. Andraos et al. [41] found that gender, age, and common life habits such as diet can all lead to changes in the concentration of TMAO in serum. These discrepant results may be also explained by differences in the laboratory detection method.

Our study has several key strengths. By capitalizing on longitudinally repeated blood sample collections within this long-term cohort, we investigated the associations between long-term changes in metabolite levels and the subsequent risk of T2DM for the first time. Additionally, the study was a prospective cohort study with more stringent inclusion criteria and subsequent high-quality follow-up. Thirdly, in addition to investigating the gut-microbiota metabolite TMAO, we also considered its three precursors, which are rarely detected repeatedly in other research. This enhances the comprehensiveness of the study design. This study has also some limitations. First, the cohort population in this study is derived from rural areas and the population diversity is limited, so it needs to be validated in a larger population. Second, the estimation of diabetes incidence may be biased because of the lack of oral glucose-tolerance tests. Third, our study lacks data on the concentration of dietary precursors, which are essential for further comparing the intake and serum levels of precursors to support our conclusions. Moreover, the assessment of dietary-related variables and other covariates included in the multivariate analysis were based on self-reports, which potentially affected the estimated risk of T2DM. Further studies with longer follow-up times and more representative samples are required to confirm our findings.

## 5. Conclusions

In conclusion, this study suggests that elevated serum levels of TMAO and choline are associated with an increased risk of T2DM among middle-aged and older adults. Furthermore, longitudinal changes in serum TMAO levels are significantly linked to the incidence of T2DM in this demographic, highlighting the importance of repeated assessments in identifying individuals at high risk. These findings imply that interventions aimed at normalizing TMAO levels, such as adopting a healthy dietary pattern, may be particularly beneficial in T2DM prevention.

## Figures and Tables

**Table 1:** Characteristics of participants according to case and control status.

Variables	Controls	Diabetes Cases	*p* *
**Characteristics at the first blood collection**			
N	1434	81	
Sex, male, n(%)	497 (34.7)	25 (30.9)	0.485
Age, y	59.09 ± 9.49	60.88 ± 8.31	0.097
BMI, kg/m^2^	24.70 ± 3.62	25.87 ± 4.15	0.005
Education, primary school or below, n(%)	568 (40.3)	39 (48.8)	0.134
Income, ≤10,000 Yuan/Year/Person, n(%)	1007 (71.6)	61 (76.3)	0.370
Family history of diabetes, n(%)	54 (3.8)	1 (1.2)	0.236
Daily average frequency of red meat	0.52 (0.21, 1.02)	0.52 (0.22, 1.02)	0.709
Daily average frequency of fish	0.04 (0.04, 0.14)	0.04 (0.04, 0.14)	0.488
Daily average frequency of dairy products	0.04 (0.00, 0.23)	0.02 (0.00, 0.23)	0.329
Daily average frequency of eggs	0.79 (0.50, 1.00)	1.00 (0.50, 1.00)	0.940
Physical activity, n(%)	407 (28.4)	15 (18.5)	0.054
Current smoker, n(%)	370 (25.8)	21 (25.9)	0.980
Current drinker, n(%)	295 (20.6)	11 (15.9)	0.351
Hypertension, n(%)	390 (27.2)	30 (37.0)	0.054
Hyperlipidemia, n(%)	144 (10.0)	13 (16.0)	0.084
Fasting plasma glucose, mmol/L	5.38 (5.06, 5.75)	6.18 (5.61, 6.65)	<0.001
Triglycerides, mmol/L	1.19 (0.86, 1.73)	1.53 (1.07, 2.52)	<0.001
Total cholesterol, mmol/L	5.06 (4.52, 5.66)	5.36 (4.63, 5.90)	0.028
HDL-cholesterol, mmol/L	1.15 (1.01, 1.32)	1.08 (0.94, 1.27)	0.009
LDL-cholesterol, mmol/L	3.26 (2.74, 3.76)	3.42 (2.86, 3.85)	0.197
TMAO, µmol/L	4.09 (2.17, 7.53)	5.08 (2.27, 8.91)	0.187
Choline, µmol/L	185.71 (130.30, 251.26)	192.67 (135.54, 258.73)	0.447
Betaine, µmol/L	106.85 (78.11, 152.87)	105.12 (74.24, 148.59)	0.472
Carnitine, µmol/L	52.28 (40.51, 72.87)	52.15 (40.75, 72.44)	0.964
**Characteristics at the second blood collectio**n			
Physical activity, n(%)	293 (20.4)	16 (19.8)	0.883
Current smoker, n(%)	336 (23.4)	20 (24.7)	0.795
Current drinker, n(%)	267 (18.6)	12 (14.8)	0.390
Hypertension, n(%)	408 (28.5)	37 (45.7)	<0.001
Hyperlipidemia, n(%)	114 (7.9)	12 (14.8)	0.029
Fasting plasma glucose, mmol/L	5.30 (4.95, 5.70)	7.75 (7.20, 8.61)	<0.001
Triglycerides, mmol/L	1.19 (0.86, 1.66)	1.41 (1.06, 2.21)	<0.001
Total cholesterol, mmol/L	4.74 (4.17, 5.36)	5.08 (4.4, 5.77)	0.003
HDL-cholesterol, mmol/L	1.39 (1.17, 1.64)	1.27 (1.09, 1.50)	0.004
LDL-cholesterol, mmol/L	2.68 (2.18, 3.2)	2.94 (2.47, 3.45)	0.004
TMAO, µmol/L	3.18 (1.59, 5.91)	4.51 (1.96, 7.02)	0.005
Choline, µmol/L	93.28 (73.94, 113.74)	93.85 (73.14, 127.66)	0.334
Betaine, µmol/L	93.38 (73.5, 120.55)	83.57 (66.88, 107.40)	0.047
Carnitine, µmol/L	52.25 (43.06, 63.16)	48.24 (40.88, 58.06)	0.026
**Changes from the baseline to second blood collections**			
ΔFasting plasma glucose, mmol/L	−0.08 (−0.41, 0.26)	1.56 (0.87, 2.75)	<0.001
ΔTriglycerides, mmol/L	−0.01 (−0.34, 0.29)	0.04 (−0.46, 0.46)	0.779
ΔTotal cholesterol, mmol/L	−0.33 (−0.74, 0.10)	−0.18 (−0.71, 0.15)	0.328
ΔHDL-cholesterol, mmol/L	0.24 (0.10, 0.39)	0.18 (0.06, 0.39)	0.117
ΔLDL-cholesterol, mmol/L	−0.57 (−0.95, −0.2)	−0.51 (−0.84, −0.03)	0.070
ΔTMAO, µmol/L	−0.90 (−3.96, 1.72)	−0.56 (−3.11, 2.68)	0.202
ΔCholine, µmol/L	−88.05 (−162.17, −28.13)	−79.2 (−165.82, −24.96)	0.774
ΔBetaine, µmol/L	−14.44 (−56.46, 20.07)	−9.05 (−54.24, 14.86)	0.971
ΔCarnitine, µmol/L	−1.35 (−20.47, 15.11)	−3.29 (−25.81, 11.98)	0.177

Data are shown as n(%), mean ± SD, or median (IQR). * Characteristics of the study participants by case-control status were compared by χ² test for categorical variables. Median values between cases and controls were compared by the median test when appropriate.

**Table 2:** Risk of T2DM according to TMAO and its precursors levels at the first collection.

	Crude Model	Model 1	Model 2	Model 3
	OR (95% CI)	OR (95% CI)	OR (95% CI)	OR (95% CI)
TMAO, µmol/L				
Q1 (≤2.19)	1.00 (Reference)	1.00 (Reference)	1.00 (Reference)	1.00 (Reference)
Q2 (2.19 to 4.16)	0.73 (0.36, 1.47)	0.71 (0.35, 1.44)	0.70 (0.34, 1.42)	0.82 (0.40, 1.70)
Q3 (4.16 to 7.64)	1.06 (0.55, 2.01)	0.99 (0.52, 1.89)	0.96 (0.50, 1.86)	1.44 (0.71, 2.93)
Q4 (≥7.64)	1.52 (0.83, 2.76)	1.39 (0.75, 2.55)	1.35 (0.73, 2.50)	3.35 (1.55, 7.26)
Per 1 SD increment	1.01 (0.82, 1.25)	1.01 (0.79, 1.29)	1.01 (0.77, 1.32)	1.10 (0.90, 1.34)
Choline, µmol/L				
Q1 (≤130.76)	1.00 (Reference)	1.00 (Reference)	1.00 (Reference)	1.00 (Reference)
Q2 (130.76 to 184.06)	1.05 (0.53, 2.08)	1.07 (0.54, 2.14)	1.03 (0.51, 2.06)	1.39 (0.62, 3.09)
Q3 (180.06 to 247.96)	1.44 (0.76, 2.72)	1.57 (0.82, 3.01)	1.49 (0.78, 2.87)	3.37 (1.41, 8.05)
Q4 (≥247.96)	1.31 (0.69, 2.49)	1.31 (0.69, 2.52)	1.24 (0.64, 2.39)	4.72 (1.47, 15.13)
Per 1 SD increment	1.02 (0.82, 1.26)	1.01 (0.81, 1.25)	0.99 (0.79, 1.26)	1.10 (0.84, 1.45)
Betaine, µmol/L				
Q1 (≤75.39)	1.00 (Reference)	1.00 (Reference)	1.00 (Reference)	1.00 (Reference)
Q2 (75.39 to 103.70)	0.85 (0.45, 1.60)	0.84 (0.44, 1.60)	0.80 (0.42, 1.53)	0.72 (0.36, 1.44)
Q3 (103.70 to 147.59)	0.90 (0.48, 1.68)	0.95 (0.51, 1.78)	0.93 (0.49, 1.76)	0.84 (0.40, 1.79)
Q4 (≥147.59)	0.79 (0.42, 1.48)	0.86 (0.46, 1.63)	0.81 (0.43, 1.54)	0.71 (0.28, 1.85)
Per 1 SD increment	0.93 (0.73, 1.17)	0.97 (0.76, 1.23)	0.94 (0.74, 1.21)	0.89 (0.61, 1.29)
Carnitine, µmol/L				
Q1 (≤40.09)	1.00 (Reference)	1.00 (Reference)	1.00 (Reference)	1.00 (Reference)
Q2 (40.09 to 51.18)	1.05 (0.55, 2.00)	1.06 (0.55, 2.03)	1.14 (0.59, 2.19)	1.03 (0.51, 2.08)
Q3 (51.18 to 70.90)	1.01 (0.53, 1.93)	1.01 (0.53, 1.93)	1.06 (0.55, 2.04)	0.82 (0.37, 1.82)
Q4 (≥70.90)	1.05 (0.56, 1.98)	1.03 (0.54, 1.96)	1.06 (0.55, 2.01)	0.49 (0.18, 1.34)
Per 1 SD increment	1.02 (0.82, 1.27)	1.02 (0.81, 1.27)	1.01 (0.81, 1.27)	0.77 (0.54, 1.11)

Abbreviation: Q, quartile; TMAO, trimethylamine-N-oxide; T2DM, type 2 diabetes mellitus. Model 1 was a logistic regression that was adjusted for age, sex, BMI, hypertension, and hyperlipidemia at the first time point. Model 2 was a logistic regression that was adjusted as for Model 1 and for physical activity, current smoking status, current drinking status, and daily average frequency of red meat, fish, dairy products and eggs at the first time point. Model 3 was a logistic regression that was adjusted as for Model 2 and for ΔTMAO or its precursors’ change.

**Table 3:** Risk of T2DM according to ΔTMAO and changes in its precursors.

	Crude Model	Model 1	Model 2	Model 3
	OR (95% CI)	OR (95% CI)	OR (95% CI)	OR (95% CI)
ΔTMAO, µmol/L				
Q1 (≤−3.95)	1.00 (Reference)	1.00 (Reference)	1.00 (Reference)	1.00 (Reference)
Q2 (−3.95 to −0.87)	1.00 (0.50, 1.98)	1.00 (0.50, 2.01)	1.01 (0.50, 2.03)	2.07 (0.93, 4.61)
Q3 (−0.87 to 1.76)	1.31 (0.68, 2.50)	1.38 (0.72, 2.66)	1.40 (0.72, 2.71)	3.59 (1.57, 8.22)
Q4 (≥1.76)	1.50 (0.80, 2.83)	1.54 (0.81, 2.93)	1.53 (0.80, 2.92)	3.69 (1.66, 8.17)
Per 1 SD increment	1.21 (0.94, 1.54)	1.21 (0.94, 1.56)	1.20 (0.94, 1.55)	1.34 (1.06, 1.71)
ΔCholine, µmol/L				
Q1 (≤−160.60)	1.00 (Reference)	1.00 (Reference)	1.00 (Reference)	1.00 (Reference)
Q2 (−160.60 to −87.52)	0.87 (0.45, 1.70)	0.97 (0.50, 1.90)	0.99 (0.50, 1.94)	1.46 (0.55, 3.89)
Q3 (−87.52 to −28.18)	1.12 (0.60, 2.09)	1.13 (0.60, 2.12)	1.16 (0.62, 2.19)	3.20 (1.11, 9.27)
Q4 (≥−28.18)	1.12 (0.60, 2.08)	1.12 (0.59, 2.10)	1.21 (0.64, 2.28)	4.56 (1.43, 14.55)
Per 1 SD increment	1.08 (0.84, 1.38)	1.08 (0.85, 1.38)	1.11 (0.86, 1.43)	1.80 (1.11, 2.90)
ΔBetaine, µmol/L				
Q1 (≤−53.66)	1.00 (Reference)	1.00 (Reference)	1.00 (Reference)	1.00 (Reference)
Q2 (−53.66 to −12.75)	1.00 (0.53, 1.91)	0.98 (0.51, 1.88)	1.00 (0.52, 1.92)	0.94 (0.43, 2.08)
Q3 (−12.75 to 19.94)	1.37 (0.75, 2.51)	1.32 (0.71, 2.42)	1.31 (0.70, 2.42)	1.15 (0.48, 2.75)
Q4 (≥19.94)	0.89 (0.46, 1.72)	0.80 (0.41, 1.56)	0.85 (0.43, 1.67)	0.70 (0.27, 1.83)
Per 1 SD increment	0.99 (0.79, 1.23)	0.95 (0.76, 1.20)	0.97 (0.77, 1.23)	0.86 (0.62, 1.20)
ΔCarnitine, µmol/L				
Q1 (≤−20.65)	1.00 (Reference)	1.00 (Reference)	1.00 (Reference)	1.00 (Reference)
Q2 (−20.65 to −1.03)	0.63 (0.34, 1.18)	0.63 (0.33, 1.18)	0.62 (0.33, 1.18)	0.42 (0.19, 0.94)
Q3 (−1.03 to 15.05)	0.82 (0.45, 1.48)	0.80 (0.44, 1.46)	0.81 (0.44, 1.48)	0.48 (0.20, 1.15)
Q4 (≥15.05)	0.65 (0.35, 1.21)	0.60 (0.32, 1.14)	0.63 (0.33, 1.19)	0.36 (0.14, 0.95)
Per 1 SD increment	0.86 (0.69, 1.08)	0.85 (0.68, 1.06)	0.87 (0.69, 1.09)	0.73 (0.51, 1.03)

Abbreviation: Q, quartile; TMAO, trimethylamine-N-oxide; T2DM, type 2 diabetes mellitus. Model 1 was a logistic regression that was adjusted for age, sex, BMI, hypertension, and hyperlipidemia at the first time point. Model 2 was a logistic regression that was adjusted as for Model 1 and for physical activity, current smoking status, current drinking status, and daily average frequency of red meat, fish, dairy products and eggs at the first time point. Model 3 was a logistic regression that was adjusted as for Model 2 and for TMAO or its precursors.

**Table 4:** Risk of T2DM according to TMAO levels at the first collection and ΔTMAO.

Group	N	Crude, OR (95% CI)	*p*	Model 1, OR (95% CI)	*p*	Model 2, OR (95% CI)	*p*
(1) low TMAO and low ΔTMAO	2/178	1.00 (Reference)	-	1.00 (Reference)	-	1.00 (Reference)	-
(2) low TMAO and high ΔTMAO	31/547	5.04 (1.20, 21.29)	0.028	4.97 (1.17, 21.06)	0.029	4.89 (1.15, 20.77)	0.031
(3) high TMAO and low ΔTMAO	32/545	5.23 (1.24, 22.02)	0.024	4.82 (1.14, 20.38)	0.033	4.70 (1.11, 19.91)	0.036
(4) high TMAO and high ΔTMAO	16/164	8.68 (1.97, 38.34)	0.004	8.23 (1.85, 36.60)	0.006	7.89 (1.77, 35.23)	0.007

Abbreviation: TMAO, trimethylamine-N-oxide; T2DM, type 2 diabetes mellitus. Participants were categorized into 4 groups based on low (≤4.159 µmol/L) or high (≥4.160 µmol/L) TMAO levels, and low (≤−0.870 µmol/L) or high (≥−0.871 µmol/L) ΔTMAO levels. Model 1 was a logistic regression that was adjusted for age, sex, BMI, hypertension, and hyperlipidemia at the first time point. Model 2 was a logistic regression that was adjusted as for Model 1 and for physical activity, current smoking status, current drinking status, and daily average frequency of red meat, fish, dairy products and eggs at the first time point.

## Data Availability

The data supporting the findings of this study are available from the Northeast China Rural Cardiovascular Health Study (NCRCHS) under license for the current study and are not publicly accessible due to restrictions.

## References

[1] Chatterjee S., Khunti K., Davies M.J. (2017). Type 2 diabetes. Lancet.

[2] Sonnenburg J.L., Bäckhed F. (2016). Diet–microbiota interactions as moderators of human metabolism. Nature.

[3] Wang Z., Klipfell E., Bennett B.J., Koeth R., Levison B.S., DuGar B., Feldstein A.E., Britt E.B., Fu X., Chung Y.-M. (2011). Gut flora metabolism of phosphatidylcholine promotes cardiovascular disease. Nature.

[4] Koeth R.A., Wang Z., Levison B.S., Buffa J.A., Org E., Sheehy B.T., Britt E.B., Fu X., Wu Y., Li L. (2013). Intestinal microbiota metabolism of L-carnitine, a nutrient in red meat, promotes atherosclerosis. Nat. Med..

[5] Ufnal M., Zadlo A., Ostaszewski R. (2015). TMAO: A small molecule of great expectations. Nutrition.

[6] Cho C.E., Caudill M.A. (2017). Trimethylamine-N-Oxide: Friend, Foe, or Simply Caught in the Cross-Fire?. Trends Endocrinol. Metab..

[7] Luo T., Guo Z., Liu D., Guo Z., Wu Q., Li Q., Lin R., Chen P., Ou C., Chen M. (2022). Deficiency of PSRC1 accelerates atherosclerosis by increasing TMAO production via manipulating gut microbiota and flavin monooxygenase 3. Gut Microbes.

[8] Zeisel S.H., Warrier M. (2017). Trimethylamine *N*-Oxide, the Microbiome, and Heart and Kidney Disease. Annu. Rev. Nutr..

[9] Subramaniam S., Fletcher C. (2018). Trimethylamine N-oxide: Breathe new life. Br. J. Pharmacol..

[10] Janeiro M.H., Ramírez M.J., Milagro F.I., Martínez J.A., Solas M. (2018). Implication of Trimethylamine N-Oxide (TMAO) in Disease: Potential Biomarker or New Therapeutic Target. Nutrients.

[11] Zheng L., Zheng J., Xie Y., Li Z., Guo X., Sun G., Sun Z., Xing F., Sun Y. (2019). Serum gut microbe-dependent trimethylamine N-oxide improves the prediction of future cardiovascular disease in a community-based general population. Atherosclerosis.

[12] Govindarajulu M., Pinky P.D., Steinke I., Bloemer J., Ramesh S., Kariharan T., Rella R.T., Bhattacharya S., Dhanasekaran M., Suppiramaniam V. (2020). Gut Metabolite TMAO Induces Synaptic Plasticity Deficits by Promoting Endoplasmic Reticulum Stress. Front. Mol. Neurosci..

[13] Kong L., Zhao Q., Jiang X., Hu J., Jiang Q., Sheng L., Peng X., Wang S., Chen Y., Wan Y. (2024). Trimethylamine N-oxide impairs beta-cell function and glucose tolerance. Nat. Commun..

[14] Zhuang R., Ge X., Han L., Yu P., Gong X., Meng Q., Zhang Y., Fan H., Zheng L., Liu Z. (2019). Gut microbe-generated metabolite trimethylamine *N*-oxide and the risk of diabetes: A systematic review and dose-response meta-analysis. Obes. Rev..

[15] Shan Z., Sun T., Huang H., Chen S., Chen L., Luo C., Yang W., Yang X., Yao P., Cheng J. (2017). Association between microbiota-dependent metabolite trimethylamine-N-oxide and type 2 diabetes. Am. J. Clin. Nutr..

[16] Kalagi N.A., Thota R.N., Stojanovski E., Alburikan K.A., Garg M.L. (2022). Association between Plasma Trimethylamine N-Oxide Levels and Type 2 Diabetes: A Case Control Study. Nutrients.

[17] Svingen G.F., Schartum-Hansen H., Pedersen E.R., Ueland P.M., Tell G.S., Mellgren G., Njølstad P.R., Seifert R., Strand E., Karlsson T. (2016). Prospective Associations of Systemic and Urinary Choline Metabolites with Incident Type 2 Diabetes. Clin. Chem..

[18] Lemaitre R.N., Jensen P.N., Wang Z., Fretts A.M., McKnight B., Nemet I., Biggs M.L., Sotoodehnia N., de Oliveira Otto M.C., Psaty B.M. (2021). Association of Trimethylamine N-Oxide and Related Metabolites in Plasma and Incident Type 2 Diabetes: The Cardiovascular Health Study. JAMA Netw. Open.

[19] Papandreou C., Bulló M., Zheng Y., Ruiz-Canela M., Yu E., Guasch-Ferré M., Toledo E., Clish C., Corella D., Estruch R. (2018). Plasma trimethylamine-N-oxide and related metabolites are associated with type 2 diabetes risk in the Prevención con Dieta Mediterránea (PREDIMED) trial. Am. J. Clin. Nutr..

[20] Sotos-Prieto M., Bhupathiraju S.N., Mattei J., Fung T.T., Li Y., Pan A., Willett W.C., Rimm E.B., Hu F.B. (2017). Association of Changes in Diet Quality with Total and Cause-Specific Mortality. N. Engl. J. Med..

[21] Wang L., Li X., Wang Z., Bancks M.P., Carnethon M.R., Greenland P., Feng Y.-Q., Wang H., Zhong V.W. (2021). Trends in Prevalence of Diabetes and Control of Risk Factors in Diabetes Among US Adults, 1999–2018. JAMA.

[22] Heianza Y., Ma W., DiDonato J.A., Sun Q., Rimm E.B., Hu F.B., Rexrode K.M., Manson J.E., Qi L. (2020). Long-Term Changes in Gut Microbial Metabolite Trimethylamine N-Oxide and Coronary Heart Disease Risk. J. Am. Coll. Cardiol..

[23] Qi S., Liu L., He S., Wang L., Li J., Sun X. (2023). Trimethylamine N-Oxide and Related Metabolites in the Serum and Risk of Type 2 Diabetes in the Chinese Population: A Case-Control Study. Diabetes Metab. Syndr. Obesity.

[24] Gao X., Liu X., Xu J., Xue C., Xue Y., Wang Y. (2014). Dietary trimethylamine N-oxide exacerbates impaired glucose tolerance in mice fed a high fat diet. J. Biosci. Bioeng..

[25] Chen S., Henderson A., Petriello M.C., Romano K.A., Gearing M., Miao J., Schell M., Sandoval-Espinola W.J., Tao J., Sha B. (2019). Trimethylamine N-Oxide Binds and Activates PERK to Promote Metabolic Dysfunction. Cell Metab..

[26] Ding L., Chang M., Guo Y., Zhang L., Xue C., Yanagita T., Zhang T., Wang Y. (2018). Trimethylamine-N-oxide (TMAO)-induced atherosclerosis is associated with bile acid metabolism. Lipids Health Dis..

[27] Yang J.J., Shu X.-O., Herrington D.M., Moore S.C., Meyer K.A., Ose J., Menni C., Palmer N.D., Eliassen H., Harada S. (2021). Circulating trimethylamine *N*-oxide in association with diet and cardiometabolic biomarkers: An international pooled analysis. Am. J. Clin. Nutr..

[28] Wang Z., Bergeron N., Levison B.S., Li X.S., Chiu S., Jia X., Koeth R.A., Li L., Wu Y., Tang W.H.W. (2019). Impact of chronic dietary red meat, white meat, or non-meat protein on trimethylamine N-oxide metabolism and renal excretion in healthy men and women. Eur. Heart J..

[29] Krishnan S., O’Connor L.E., Wang Y., Gertz E.R., Campbell W.W., Bennett B.J. (2021). Adopting a Mediterranean-style eating pattern with low, but not moderate, unprocessed, lean red meat intake reduces fasting serum trimethylamine N-oxide (TMAO) in adults who are overweight or obese. Br. J. Nutr..

[30] David L.A., Maurice C.F., Carmody R.N., Gootenberg D.B., Button J.E., Wolfe B.E., Ling A.V., Devlin A.S., Varma Y., Fischbach M.A. (2014). Diet rapidly and reproducibly alters the human gut microbiome. Nature.

[31] Wang Z., Roberts A.B., Buffa J.A., Levison B.S., Zhu W., Org E., Gu X., Huang Y., Zamanian-Daryoush M., Culley M.K. (2015). Non-lethal Inhibition of Gut Microbial Trimethylamine Production for the Treatment of Atherosclerosis. Cell.

[32] Falony G., Vieira-Silva S., Raes J. (2015). Microbiology Meets Big Data: The Case of Gut Microbiota–Derived Trimethylamine. Annu. Rev. Microbiol..

[33] Wang S., Xia G.-H., He Y., Liao S.-X., Yin J., Sheng H.-F., Zhou H.-W. (2016). Distribution characteristics of trimethylamine N-oxide and its association with gut microbiota. J. South Med. Univ..

[34] Farahbod K., Slouha E., Gerts A., Rezazadah A., Clunes L.A., Kollias T.F. (2024). The Effects of Diet Intervention on the Gut Microbiota in Type 2 Diabetes Mellitus: A Systematic Review. Cureus.

[35] Chen L., Liu B., Ren L., Du H., Fei C., Qian C., Li B., Zhang R., Liu H., Li Z. (2023). High-fiber diet ameliorates gut microbiota, serum metabolism and emotional mood in type 2 diabetes patients. Front. Cell. Infect. Microbiol..

[36] Luo W., Zhou J., Yang X., Wu R., Liu H., Shao H., Huang B., Kang X., Yang L., Liu D. (2022). A Chinese medical nutrition therapy diet accompanied by intermittent energy restriction alleviates type 2 diabetes by enhancing pancreatic islet function and regulating gut microbiota composition. Food Res. Int..

[37] Cho E., Zeisel S.H., Jacques P., Selhub J., Dougherty L., Colditz G.A., Willett W.C. (2006). Dietary choline and betaine assessed by food-frequency questionnaire in relation to plasma total homocysteine concentration in the Framingham Offspring Study. Am. J. Clin. Nutr..

[38] Rath S., Rox K., Bardenhorst S.K., Schminke U., Doerr M., Mayerle J., Frost F., Lerch M.M., Karch A., Broenstrup M. (2021). Higher Trimethylamine-N-Oxide Plasma Levels with Increasing Age Are Mediated by Diet and Trimethylamine-Forming Bacteria. mSystems.

[39] Ufnal M., Jazwiec R., Dadlez M., Drapala A., Sikora M., Skrzypecki J. (2014). Trimethylamine-N-oxide: A carnitine-derived metabolite that prolongs the hypertensive effect of angiotensin II in rats. Can. J. Cardiol..

[40] Nie J., Xie L., Zhao B.-X., Li Y., Qiu B., Zhu F., Li G.-F., He M., Wang Y., Wang B. (2018). Serum Trimethylamine N-Oxide Concentration Is Positively Associated with First Stroke in Hypertensive Patients. Stroke.

[41] Andraos S., Lange K., Clifford S.A., Jones B., Thorstensen E.B., Kerr J.A., Wake M., Saffery R., Burgner D.P., O’Sullivan J.M. (2020). Plasma Trimethylamine N-Oxide and Its Precursors: Population Epidemiology, Parent-Child Concordance, and Associations with Reported Dietary Intake in 11- to 12-Year-Old Children and Their Parents. Curr. Dev. Nutr..

